# An Assessment of the Effect of Rotenone on Selected Non-Target Aquatic Fauna

**DOI:** 10.1371/journal.pone.0142140

**Published:** 2015-11-05

**Authors:** Tatenda Dalu, Ryan J. Wasserman, Martine Jordaan, William P. Froneman, Olaf L. F. Weyl

**Affiliations:** 1 Department of Zoology and Entomology, Rhodes University, Grahamstown, Eastern Cape, South Africa; 2 South African Institute for Aquatic Biodiversity, Grahamstown, Eastern Cape, South Africa; 3 Centre for Invasion Biology, South African Institute for Aquatic Biodiversity, Grahamstown, Eastern Cape, South Africa; 4 Scientific Services Department, CapeNature, Stellenbosch, Western Cape, South Africa; Bournemouth University, UNITED KINGDOM

## Abstract

Rotenone, a naturally occurring ketone, is widely employed for the management of invasive fish species. The use of rotenone poses serious challenges to conservation practitioners due to its impacts on non-target organisms including amphibians and macroinvertebrates. Using laboratory studies, we investigated the effects of different rotenone concentrations (0, 12.5, 25, 37.5, 50, 100 μg L^-1^) on selected invertebrate groups; Aeshnidae, Belostomatids, Decapods, Ephemeroptera, Pulmonata and zooplankton over a period of 18 hours. Based on field observations and body size, we hypothesized that Ephemeropterans and zooplankton would be more susceptible to rotenone than Decapods, Belostomatids and snails. Experimental results supported this hypothesis and mortality and behaviour effects varied considerably between taxa, ranging from no effect (crab *Potamonuates sidneyi*) to 100% mortality (*Daphnia pulex* and *Paradiaptomus lamellatus*). Planktonic invertebrates were particularly sensitive to rotenone even at very low concentrations. Future research should investigate the recovery time of invertebrate communities after the application of rotenone and conduct field assessments assessing the longer term effects of rotenone exposure on the population dynamics of those less sensitive organisms.

## Introduction

Globally, biological invasions are becoming increasingly problematic and are considered potential drivers of biodiversity loss and in some instances are associated with economic threats [1, 2, 3]. Conservation practitioners are increasingly implementing better methods to control the spread of invasive species, particularly in ecologically sensitive areas or areas of high conservation priority [4, 5, 6]. In freshwater ecosystems, biodiversity declines are of particular concern as these environments are considered to be among some of the most threatened systems, with invasions contributing to their deterioration [2, 7]. Non-native fishes contribute considerably in this regard [8]. For the control of non-native fishes, eradication is often considered an option due to the effectiveness of available piscicides [9]. Rotenone, a naturally occurring ketone, is one such piscicide which has been successfully used for fish biocontrol around the world [6, 9–11].

Rotenone is derived from the roots of plants belonging to the family *Leguminosae* including the jewel vine (*Derris* spp.) and lacepod (*Lonchocarpus* spp.), that grow in Oceania, Central and South America and south-east Asia [12, 13]. Over the last 150 years it has also been used extensively as a commercial insecticide to deter slugs and snails from garden vegetables [12] and for more than 70 years, rotenone has been an important tool both for recreational fisheries management and, more recently, for the restoration of native fish species [6, 9, 10, 14, 15].

The use of rotenone, however, poses a challenge to conservation practitioners because detrimental impacts to non-target freshwater organisms such as amphibians [16] and invertebrates [17] have been reported. Impacts on aquatic invertebrates tend to be highly variable and taxon-specific, making the environmental impacts of proposed rotenone operations difficult to predict [17]. Woodford et al. [18] for example, observed that the immediate impact of rotenone operations on the Rondegat River, South Africa, appeared to have been most severe on the Ephemeropterans, which were among the quickest to respond to rotenone in the water through mass drift and declined significantly in abundance following treatment. While it is recognized that rotenone is likely to affect numerous aquatic taxa, there are few studies that have assessed such impacts [15–18] and as a result the use of rotenone remains contentious as its effect on non-target biota are still largely unknown [6, 9, 11, 17, 19–21].

In headwater streams in South Africa’s Cape Floristic Region, an area of high freshwater fish and invertebrate biodiversity and endemism [22], the primary threat to aquatic biota in the region is considered to be predation by and competition with non-native fish species [6, 23]. While non-native fish eradication using rotenone is currently considered the most appropriate conservation intervention [6, 24], the public as well as the Department of Water Affairs (the South African regulatory authority) have expressed concern on the potential impact of river treatments on non-target organisms (see [6]). These concerns have resulted in delays in the approval of rotenone treatments of several rivers and off-channel impoundments (N.D. Impson, Scientist, CapeNature). To provide guidance for the use of rotenone for future interventions native fish restoration projects [6], the response of insect communities following rotenone treatments are being monitored [18, 25]. A major constraint in applying the results of a field study to predict impacts on other systems, is that treatment concentrations differ between fish species [26] and there are few studies describing the effects of rotenone on invertebrates [10, 15, 27]. Further, laboratory studies typically use exposure durations that are substantially longer than those used in rotenone treatments [27]. This leads to considerable uncertainty regarding taxon-specific effects and susceptibility of invertebrates to rotenone in different habitats [9, 11, 19, 28].

**Fig 1:**
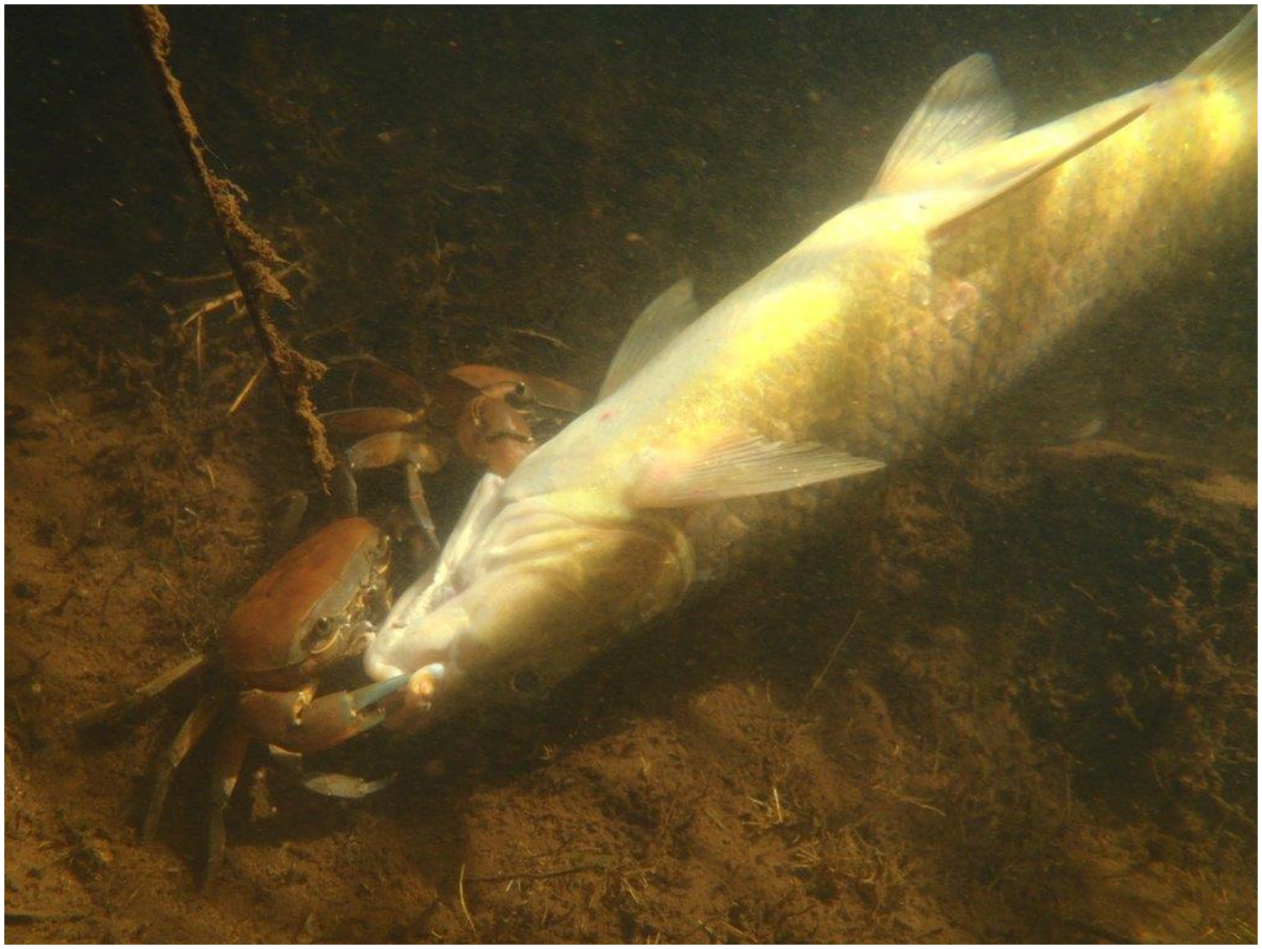
*Potamonuates* sp. feeding on a dead fish in the Rondegat River, South Africa soon after rotenone application (photo by Bruce R. Ellender).

For this reason, we assessed the short-term responses of rotenone exposure on selected aquatic invertebrates, using concentrations and exposure durations typically used in river treatments [29, 30]. To the best of our knowledge, the effect of rotenone concentration on invertebrate taxa has only been assessed to a small extent (see [15]) using field application protocol treatments and exposure times in the laboratory. Our experimental design followed standard piscicide application protocols [12, 15, 29, 30] and this resulted in a unique opportunity to compare the results from short-term exposure experiments to field observations i.e. the Rondegat River. Field studies conducted before and after rotenone treatment in many parts of the world have noted a decline in some members of the macroinvertebrate community [9, 11, 13, 19–21, 25]. Observations in Rondegat River, during rotenone treatments also indicated that other invertebrates, such as *Potamonuates sidneyi*, appeared to be unaffected and were observed feeding on fish that had succumbed to the rotenone during the treatment (Fig 1). Therefore, our aim was to experimentally determine the effects of rotenone exposure on representative aquatic insect, crustacean and gastropod taxa. We hypothesized that 1.) based on field observations, Ephemeropterans would be more susceptible to rotenone than Aeshnids, Decapods, Belostomatids and snails [18, 25], and 2.) Copepods, Ostracods and Daphniids would be particularly susceptible given their low tolerance for environmental toxicants [31–33].

## Material and Methods

### Aquatic invertebrates sampling

Collection permits were obtained from Eastern Cape Department of Economic Development and Environmental Affairs (DEDEA permit no. CRO 3/12CR, CRO4/12CR, CRO 12/14CR and CRO 13/14CR) and ethical clearance was obtained from the South African Institute for Aquatic Biodiversity (reference no. 2014/01). Representative species of key selected taxa were collected from natural (i.e. unimpacted) freshwater environments of the Eastern Cape of South Africa. These representatives included the freshwater crab *Potamonuates sidneyi* (Decapoda, size range 1–1.5 cm), diving beetle *Diplonychus capensis* (Belostomatidae, size range 0.8–1 cm), mayfly *Baetis harrisonii* (Ephemeroptera, size range 0.7–0.9 cm), dragon fly *Anax imperator* (Aeshnidae, size range 2–2.4 cm), freshwater snail *Physa acuta* (Physidae, size range 0.4–0.7 cm), *Cypricercus* sp. (Ostracoda, size range 0.2–0.4 cm), *Paradiaptomus lamellatus* (Copepoda, size range 0.3–0.4 cm) and water flea *Daphnia pulex* (Cladocera size range 0.1–0.2 cm). Crabs, diving beetles, dragon flies, mayflies and snails were collected using standard SASS kick nets. Ostracods, copepods and water flea were collected by towing a zooplankton net (50 cm Ø, 64 μm mesh) through the water column. All collected invertebrates were sorted in the field and placed in separate labelled buckets for transportation back to the laboratory. Water was collected from the sampling locations for the invertebrate holding tanks and for conducting the experiments. All animals were acclimatized at 20 ± 0.5°C for a period of 48 hours under constant aeration, prior to experimentation under a 24 h light photoperiod cycle in temperature controlled environmental rooms.

### Experimental design

The Organisation for Economic Co-operation and Development (OECD) guidelines for acute toxicity studies were followed for the experimental procedures, with four replicates (n = 4) run for each invertebrate group at six rotenone concentrations [34]. Within each replicate, 5 individuals were placed in each experimental container. This was done for all experimental taxa except for *P*. *sidneyi*, which were observed to interact aggressively (fighting, killing and cannibalism) when multiple individuals were placed in single containers during preliminary trials. To avoid confounding effects from deaths not related to rotenone exposures, *P*. *sidneyi* were individually housed within each container, with 10 replicates employed for each of the experimental rotenone concentrations.

A fresh stock solution of 0.150 mg rotenone L^−1^ was prepared on the day of each experimental trial from the commercial piscicide CFT Legumine ^®^ (5% active rotenone) which is registered in the United States (EPA Registration number 75338–2). This solution was prepared and diluted using filtered (through a 20 μm mesh sieve) water from the sample location to make up the five treatment concentrations: 12.5, 25, 37.5, 50 and 100 μg L^−1^. In addition, a no rotenone (0 μg L^−1^) treatment was employed using the same filtered water which served as a control. The experimental design included rotenone treatment concentrations (50 and 37.5 μg L^-1^) and duration (6-hour) used during the smallmouth bass *Micropterus dolomieu* eradication in the Rondegat River South Africa [25, 26, 30]. Additional concentrations (12.5–100 μg L^−1^) were included based on recommended concentrations for more susceptible (e.g. rainbow trout *Oncorhynchus mykiss*) and more tolerant fish species such as carp (*Cyprinus carpio*) and bullheads (*Ameiurus* spp.) by Finlayson et al., [29]. For each taxon, individuals were placed in a glass container filled with 500 ml of each rotenone concentration, receiving no supplementary aeration during the experiment [34]. After introductions of the taxa into the rotenone solution, invertebrates were observed every hour for the first 6 hrs, and again after 18 hrs, at which point the experiment was terminated. Mortality, defined as the cessation or absence of movement after repeated tactile stimulation/prodding [27], was recorded after each observation period. A number of behavioural traits associated with rotenone toxicity, depending on the invertebrate taxa, were also assessed at each time interval, such as loss of equilibrium, location in the jar (surface, middle, bottom, position on glass surface), swimming and cessation of movement (i.e. death).

### Data analysis

To test for time and treatment effects on mortality rates for the selected invertebrate groups we employed a 2×2 Permutation Analysis of Variance (PERMANOVA; [35]), based on Euclidean dissimilarities as a distance measure, using rotenone concentration and time as factors. Using this analysis, differences in mortality were assessed at the treatment, time, and the treatment × time level (interactions between treatment and time). This analysis was conducted using PRIMER v6 add-on package PERMANOVA+ [36], using 9999 permutations [37], with significant terms investigated using a posteriori pair-wise comparisons with the PERMANOVA *t* statistic [36].

The dependence of the distribution of individuals displaying behavioural traits associated with a 6 hour exposure (representing field exposure) to different rotenone concentrations (0, 12.5, 25, 37.5, 50 and 100 μg L^-1^) was tested for *A*. *imperator*, *B*. *harrisonii*, *Cypricercus* sp., *D*. *capensis* and *P*. *acuta* using χ^2^ contingency tables in Microsoft Excel 2007. For this test, 6 concentrations × 3 traits (*B*. *harrisonii*, *Diplonychus capensis* and *Anax imperator*) and 6 concentrations × 4 traits (*P*. *acuta*, and *Cypricercus* sp.) were used for the cross-tabulations (see S1 Table).

## Results

**Fig 2:**
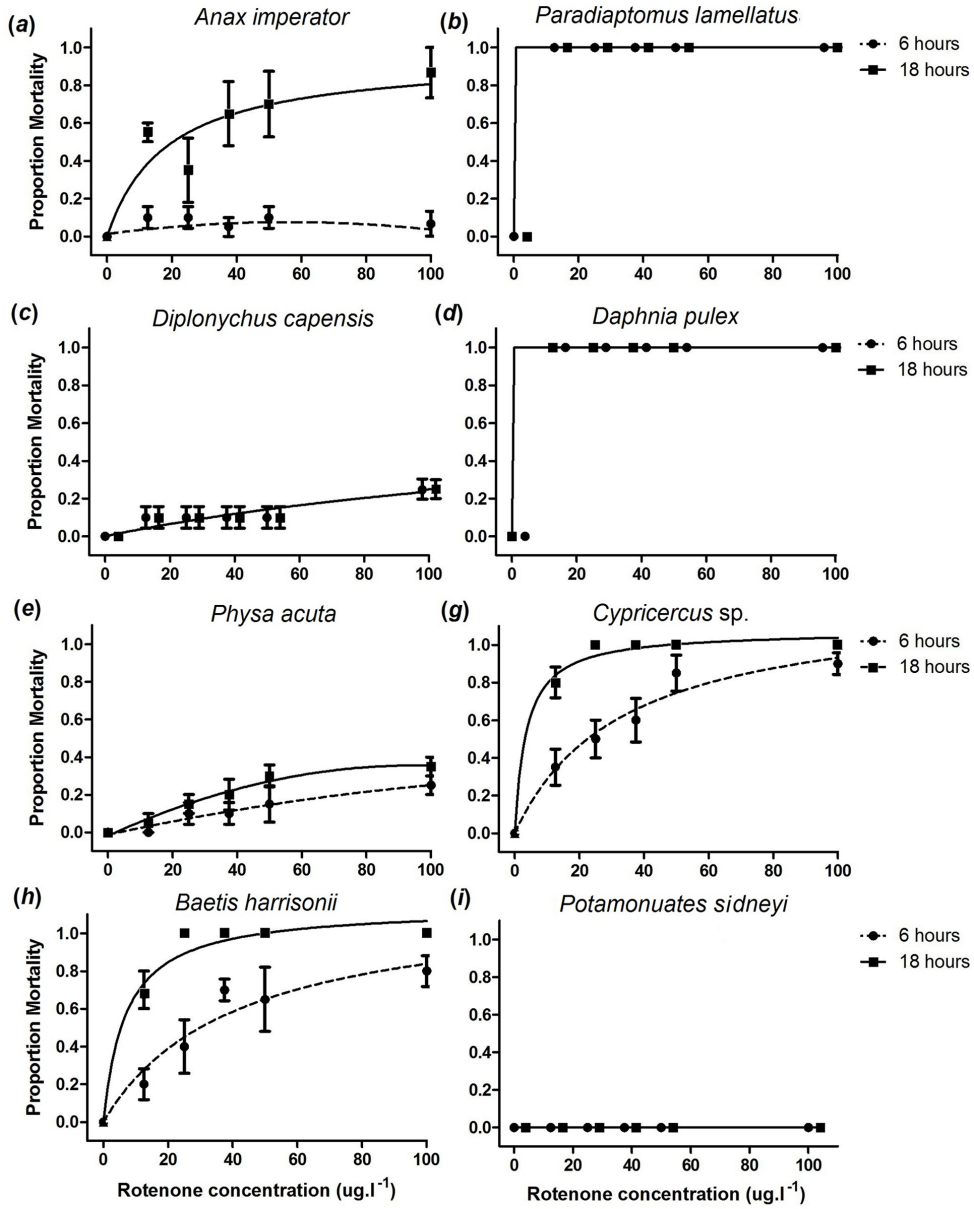
Effect of different rotenone concentrations on selected invertebrate fauna at 6 hrs and 18 hrs.

No mortalities were observed in the control treatment for any of the taxa (S1 Table). *Paradiaptomus lamellatus* and *D*. *pulex* were the most rapidly affected species, as all died within the first hour of exposure at each concentration (Fig 2b and 2d). For the remaining groups (Fig 2), exposure time and concentration became more important given that there were significant effects of both variables and interactions between the variables on mortality, with the exception of *P*. *acuta* at the Concentration × Time level and *D*. *capensis*, where Time and Concentration × Time had no significant effect (Table 1). For *P*. *sidneyi*, no mortalities were observed at any of the rotenone concentrations (Fig 2i). There were significant differences (*p* < 0.05) in the rate of mortality in most invertebrate groups across different rotenone concentrations (Table 1). The dragonflies *A*. *imperator*, *B*. *harrisonii*, *Cypricercus* sp. and *P*. *acuta* mortality rates were found to differ among time and concentrations (*p*-*perm* < 0.05), while the combined effect of concentration and time was found to have a significant effect on *A*. *imperator*, *B*. *harrisonii* and *Cypricercus* sp. mortality rates (Table 1). Using pairwise comparisons, mortality rates was found to differ significantly (*p*-*perm* < 0.05) between the two time intervals, 6 and 18 hours for selected invertebrates, excluding *D*. *capensis*, which had similar mortality rates (*p*-*perm* = 1; S2 Table). Pairwise comparisons for *A*. *imperator*, *B*. *harrisonii*, *Cypricercus* sp., *D*. *capensis* and *P*. *acuta* at different concentrations and time intervals are highlighted in the S2 Table. *Anax imperator* mortality rates at 0 μg L^-1^ was found to differ with all concentrations, while other concentrations were not significantly different across concentrations using pairwise comparisons (S2 Table).

**Table 1:** PERMANOVA test results of the effects of rotenone concentration (0–100 μg L^-1^) and time (6 and 18 hrs) on the behavioural traits of selected invertebrates. Significant differences at *p*-*perm* < 0.05 are indicated in bold. Abbreviations; df = degrees of freedom, MC = Monte Carlo, MS = mean squares, perm = permutation.

Source	df	MS	Pseudo-F	*p*-*perm*	*P*(MC)
***Anax imperator***					
Concentration	5	0.1720	3.1918	**0.0199**	**0.0179**
Time	1	2.0833	38.6600	**0.0001**	**0.0001**
Concentration×Time	5	0.1213	2.2515	**0.0302**	**0.0456**
Residual	36	0.0539			
***Baetis harrisonii***					
Concentration	5	0.9633	42.2930	**0.0001**	**0.0001**
Time	1	1.2033	52.8290	**0.0001**	**0.0001**
Concentration×Time	5	0.0853	3.7463	**0.0075**	**0.0078**
Residual	36	0.0228			
***Cypricercus* sp.**					
Concentration	5	0.9993	58.0260	**0.0001**	**0.0001**
Time	1	0.8533	49.5480	**0.0001**	**0.0001**
Concentration×Time	5	0.0873	5.0710	**0.0012**	**0.0016**
Residual	36	0.0172			
***Diplonychus capensis***					
Concentration	5	0.0513	4.8632	**0.0020**	**0.0015**
Time	1	0.0000	0.0000	1.0000	1.0000
Concentration×Time	5	0.0000	0.0230	0.7892	
Residual	36	0.0106			
***Physa acuta***					
Concentration	5	0.1055	8.8326	**0.0001**	**0.0001**
Time	1	0.0675	5.6512	**0.0219**	**0.0226**
Concentration×Time	5	0.0055	0.4605	0.8064	0.8028
Residual	36	0.0119			

**Fig 3:**
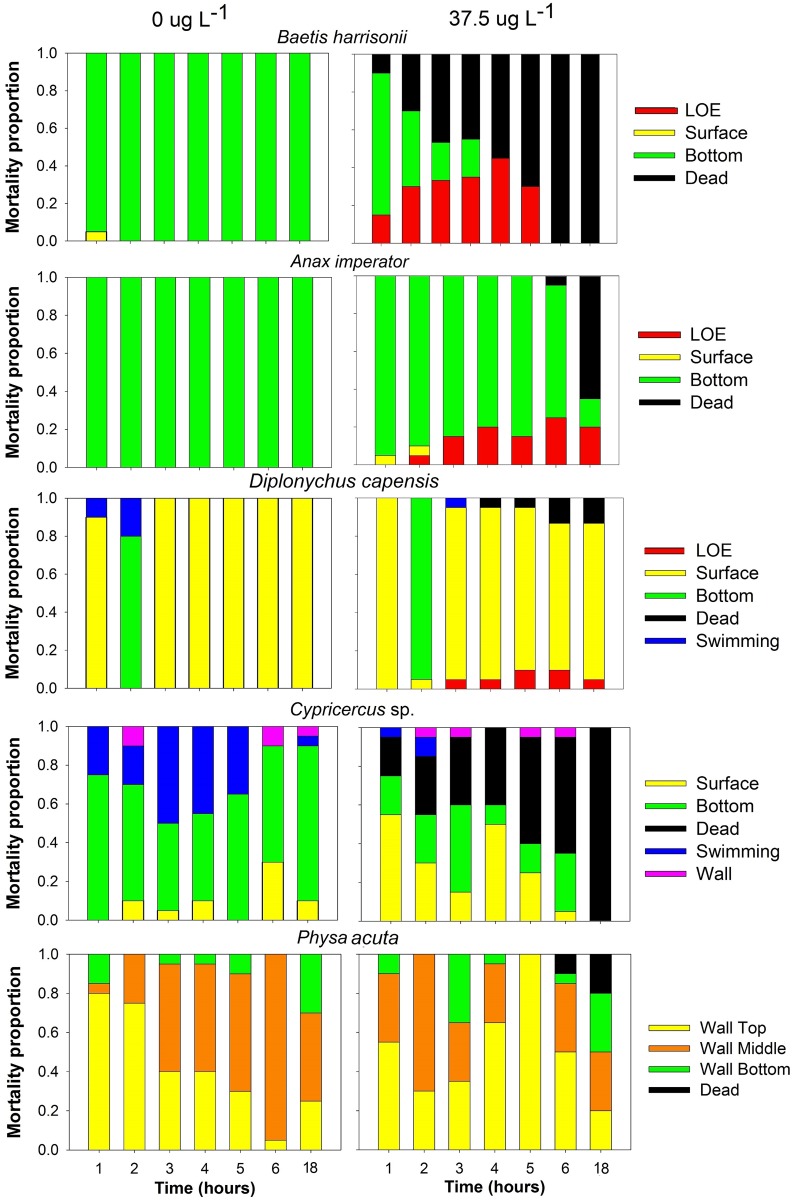
Behaviour of selected invertebrate taxa over an 18 hour exposure period at rotenone concentrations of 0 and 37.5 μg L^-1^.

Invertebrate behaviour was affected by rotenone concentration and exposure time (Fig 3, S1 Table). A graphical illustration of the proportion of invertebrates exhibiting each behavioural trait (e.g. loss of equilibrium, location in the jar, swimming and cessation of movement) recorded hourly at control (0 μg L^-1^) and field rotenone concentration (37.5 μg L^-1^) are presented in Fig 3.

Chi-square contingency table comparisons at the six hour interval demonstrated that the distribution of individuals exhibiting each behavioural trait was independent of rotenone concentration for *A*. *imperator* (χ^2^ = 5.201, df = 10, *p* = 0.877), *Cypricercus* sp. (χ^2^ = 5.201, df = 10, *p* = 0.877), *D*. *capensis* (χ^2^ = 2.668, df = 10, *p* = 0.988) and *P*. *acuta (*χ^2^ = 8.476, df = 15, *p* = 0.903). The behaviour of *B*. *harrisonii*, was however dependent on rotenone concentration (χ^2^ = 20.84, df = 10, *p* = 0.02).

## Discussion

The toxicity of rotenone to invertebrates varied considerably among taxa ranging from no effect (*P*. *sidneyi*) to 100% mortality even at low concentrations (*D*. *pulex* and *P*. *lamellatus*) (Fig 2). Such information is important for managers and conservation practitioners using piscicides as management tools, particularly in areas of high invertebrate endemicity, because it allows for the evaluation of the risk to non-target biota during rotenone treatments intended to eradicate fishes when alternative methods are either not cost effective, not feasible or ineffective [13, 27, 29]. In addition, the current paper adds potamonautid crab responses to the available knowledge on the impacts of rotenone applications on non-target biota, which have largely focused on insects [16, 17, 25].

Typical rotenone applications for river treatments last for 4–8 hrs at concentrations that are at least twice the experimentally-derived minimum effective dose (MED) that result in 100% mortality of the target organism in a 4 h period [29]. The Rondegat River for example, was treated twice, one year apart, using rotenone concentrations of 50 μg L^−1^ (4 × MED for *M*. *dolomieu*, Jordaan and Weyl [26]) and 37.5 μg L^−1^ for 6 hrs (3 × MED), respectively [30]. The first 50 μg L^−1^ rotenone treatment resulted in a substantial invertebrate drift event and a large depletion of gill-respiring EPT (Ephemeroptera, Plecoptera and Trichoptera) taxa but not in plastron respiring groups such as the Corixidae [18]. The second, more conservative 37.5 μg L^−1^ rotenone treatment also resulted in similar depletion of EPT taxa [25] suggesting mortality at significantly lower concentrations than those used during the treatment. These field observations are consistent with laboratory toxicity trials which have found the Ephemeroptera to be particularly vulnerable to rotenone exposure [15, 17, 38, 39] and the current laboratory study which demonstrated that *B*. *harrisonii* was more sensitive (100% mortality at > 25 μg L^−1^ rotenone concentration) than other insect taxa (Figs 2 and 3). In addition, this was the only test species of which differences in behaviour were observed at the 6 hr exposure treatment.

Benthic Aeshnid *A*. *imperator*, gastropod *P*. *acuta* and decapod *P*. *sidneyi* demonstrated the lowest mortality rates (Fig 2). Of particular interest was that for the decapod *P*. *sidneyi*, no deaths were observed at experimental concentrations which were as high as 100 μg L^−1^. These observations are similar to field [11, 17, 25, 40, 41] and laboratory observations reported in the literature [27, 42, 43, 44]. In acute toxicity tests, Chandler and Marking [42] for example, demonstrated that the tolerance of Dragonfly niaid *Macromia* sp., gastropods including *Physa pomilia* and the freshwater prawn *Palaemonetes kadiakensis* far exceeded the tolerances of the water flea *D*. *pulex* and Ostracod *Cypridopsis* sp. Similarly, our data suggest that potamonautid crab responses are consistent with observations on other decapods such as crayfish. For example, Vinson et al. [17] noted that benthic organisms were less sensitive to rotenone when compared to pelagic organisms. Melaas et al. [11] also highlighted that during piscicide applications, benthic organisms can seek refuge in organic sediments. However, as the current experiment did not include sediment, the use of refugia does not adequately explain low impacts on benthic taxa (e.g. *A*. *imperator*, *P*. *acuta* and *P*. *sidneyi*). Similarly, Recsetar and Bonar [44] observed 0% mortality in crayfish at recommended rotenone dosages and Wujtewicz et al. [43] demonstrated that rotenone concentrations required to kill crayfish *Procambrus acutus* (4 mg L^−1^) were > 20× higher than those resulting in 100% mortality in white perch *Morone americana* (0.15 mg L^−1^). The absence of any mortality in crabs (observed in this study) and crayfish [43, 44] can likely be attributed to the open circulatory system in Decapods [10, 44]. Öberg [10] highlighted that rotenone cause’s death at a cellular level and not at the water–blood interface. Hence, one will expect death by tissue anoxia to take longer and require higher rotenone concentrations for decapods [45]. While additional range testing would be necessary to determine lethal concentrations of rotenone to *P*. *sidneyi*, our data suggest that river treatments typically using concentrations <100 μg L^−1^ are unlikely to impact on *Potamonautes* spp. populations. This observation is supported by field observations of *Potamonuates* sp. consuming dead fish in the Rondegat River during rotenone treatments (Fig 1).

Our findings on high mortalities for zooplankton (e.g. *D*. *pulex* and *P*. *lamellatus*) at relatively low rotenone concentrations (25 μg L^−1^) are consistent with field research that has focused specifically on zooplankton responses to rotenone use [11, 13, 46, 47, 48]. Recent literature e.g. [9, 11, 44, 49–55] have reported short-term extirpation of zooplankton after rotenone application, followed by a relatively rapid recovery of zooplankton communities. Examples include two lakes in Jasper National Park, Canada [47], Upper Karori Reservoir, New Zealand [46], Fern [52] and Diamond [55] Lakes in the USA, and Lakes Salmo and Alm in Sweden [53]. The high mortalities observed for the zooplankton species during this investigation are therefore, not unexpected (Fig 2). As much of the crustacean zooplankton produce resting or dormant eggs that reside and persist in the sediment through periods of unfavourable environmental conditions [49], the presence of an “egg reservoir” may have assisted in the lack of long term effects of rotenone on zooplankton in previous field studies [11, 13, 46]. In addition, other post-treatment factors such as lack of fish predation pressure and shifting invertebrate community dynamics likely aided in zooplankton recovery in these studies [11, 13, 46]. While this suggests that zooplankton communities may be largely impervious to effects of rotenone treatment, this resilience would likely be dependent on the presence of healthy resting egg propagules within a system. In lotic systems i.e. rivers, re-colonisation from upstream is likely also to be rapid. Impacts on zooplankton communities are however mostly a concern for treatments of natural lentic waterbodies that have endemic zooplankton communities [49, 50]. In southern Africa, zooplankton communities in such environments are poorly studied and there is a need for further studies on aspects of the reproductive biology of certain zooplankton groups.

The primary action of rotenone is to block important biochemical pathways of cell metabolism, via the re-oxidation of nicotinamide adenine dinucleotide (NADH), and thereby inhibiting respiration at the cellular level [10, 56]. Fish are particularly susceptible to rotenone due to the efficiency of entry of the toxin through their gills [10], but other taxa such as gill-respiring aquatic organisms [16] and aquatic invertebrates that absorb rotenone through their tracheal gills and cuticles have also been shown to be susceptible to exposure to it, as highlighted in this and other studies e.g. [9, 13, 19, 27, 57]. In the current study, taxa that have membranes specific for gas exchange (e.g. *B*. *harrisonii*, *Cypricercus* sp., *D*. *pulex* and *P*. *lamellatus*), which have gill-like lamellae were noticeably more impacted by rotenone than those that had different breathing structures i.e. the plastron breathers (e.g. *A*. *imperator* and *D*. *capensis*).

To fully understand and minimize rotenone effects on non-target taxa, more laboratory studies should be carried to determine survivorship of several macroinvertebrate and zooplankton taxa at different stages of their development and assess how they are impacted by rotenone. In addition, assessments of whether animals would recover after exposure times would be useful and were not conducted in the present study. Latent toxicity effects may have resulted in higher mortalities in certain groups than those observed at the end of the experiment. This is a relevant consideration as in field exposures, rotenone is neutralised after a given period of time [18, 25]. The latent effects of invertebrates exposed to rotenone after neutralisation is, therefore, a consideration that is yet to be assessed for invertebrates of the region. Since most aquatic insects have terrestrial life forms, re-colonisation levels in aquatic systems treated with rotenone are likely high. While no analytical confirmation of rotenone exposure levels were assessed in the laboratory, the procedure employed was reflective of field trials where theoretical levels are determined based on point source introductions of rotenone stock solution at a known concentration [18, 30]. As such, controlled field studies such as those by Melaas et al. [11], Blakely et al. [13] and Beal and Anderson [46] assessing *in situ* effects of rotenone on macroinvertebrate and zooplankton communities should be conducted to give us an understanding of which particular taxa may be vulnerable in the long term.

## Supporting Information

S1 Table Mean proportion (± standard error) Behaviour traits of selected invertebrates after exposure to different rotenone concentrations (0–100 μg L^-1^) and time intervals (1–18 hours). (XLSX)

S2 Table Pairwise comparisons for mortality rates at different concentrations (0–100 μg L^-1^) at 6 and 18 hours for selected invertebrate groups. Significant differences at *p*-*perm* < 0.05 are indicated in bold. Abbreviation: MC = Monte Carlo, perm = permutation, t = test statistic.
(DOCX)
