# Supplementary material for: An Assessment of the Effect of Rotenone on Selected Non-Target Aquatic Fauna
Source: PLoS One. 2015 Nov 5;10(11):e0142140. doi: 10.1371/journal.pone.0142140 (PMC4634929; doi:10.1371/journal.pone.0142140)
Supplement: S2 Table — Significant differences at p-perm < 0.05 are indicated in bold. Abbreviation: MC = Monte Carlo, perm = permutation, t = test statistic. (DOCX) [file pone.0142140.s002.docx]

**S2 Table.** Pairwise comparisons for mortality rates at different concentrations (0 – 100 µg L^-1^) at 6 and 18 hours for selected invertebrate groups. Significant differences at *p*-*perm* < 0.05 are indicated in bold. Abbreviation: MC = Monte Carlo, perm = permutation, t = test statistic

| **Groups** | ***Anax imperator*** | | | ***Baetis harrisonii*** | | | ***Cypricercus* sp.** | | | ***Diplonychus capensis*** | | | ***Physa acuta*** | | |
| --- | --- | --- | --- | --- | --- | --- | --- | --- | --- | --- | --- | --- | --- | --- | --- |
|  | **t** | ***p*-*perm*** | ***P*(MC)** | **t** | ***p*-*perm*** | ***P*(MC)** | **t** | ***p*-*perm*** | ***P*(MC)** | **t** | ***p*-*perm*** | ***P*(MC)** | **t** | ***p*-*perm*** | ***P*(MC)** |
|  |  |  |  |  |  |  |  |  |  |  |  |  |  |  |  |
| **Time** |  |  |  |  |  |  |  |  |  |  |  |  |  |  |  |
| 6, 18 | 6.2177 | **0.0001** | **0.0001** | 7.2684 | **0.0001** | **0.0001** | 7.0391 | **0.0001** | **0.0001** | 0.0000 | 1.0000 | 1.0000 | 2.3772 | **0.0246** | **0.0230** |
|  | |  |  |  |  |  |  |  |  |  |  |  |  |  |  |
| **Concentrations** | |  |  |  |  |  |  |  |  |  |  |  |  |  |  |
| 0, 12.5 | 8.5105 | **0.0002** | **0.0001** | 8.8780 | **0.0003** | **0.0001** | 9.1393 | **0.0003** | **0.0001** | 2.4495 | 0.0769 | **0.0324** | 1.0000 | 0.4901 | 0.3405 |
| 0, 25 | 2.4962 | **0.0202** | **0.0281** | 9.8995 | **0.0001** | **0.0001** | 15.0000 | **0.0001** | **0.0001** | 2.4495 | 0.0749 | **0.0310** | 3.2733 | **0.0158** | **0.0061** |
| 0, 37.5 | 3.9337 | **0.0028** | **0.0017** | 29.4450 | **0.0002** | **0.0001** | 13.8560 | **0.0003** | **0.0001** | 2.4495 | 0.0780 | **0.0300** | 3.0000 | **0.0147** | **0.0100** |
| 0, 50 | 4.3818 | **0.0017** | **0.0006** | 9.6614 | **0.0002** | **0.0001** | 19.3230 | **0.0003** | **0.0001** | 2.4495 | 0.0730 | **0.0314** | 4.0249 | **0.0023** | **0.0024** |
| 0, 100 | 2.8983 | **0.0217** | **0.0126** | 22.0450 | **0.0003** | **0.0001** | 32.9090 | **0.0002** | **0.0001** | 7.0711 | **0.0007** | **0.0001** | 8.4853 | **0.0002** | **0.0001** |
| 12.5, 25 | 1.0215 | 0.3542 | 0.3210 | 3.2205 | **0.0109** | **0.0073** | 2.1776 | **0.0458** | 0.0527 | 0.0000 | 1.0000 | 1.0000 | 2.1909 | 0.0703 | **0.0484** |
| 12.5, 37.5 | 0.2582 | 0.8395 | 0.8029 | 7.6026 | **0.0001** | **0.0001** | 2.6349 | **0.0270** | **0.0210** | 0.0000 | 1.0000 | 1.0000 | 2.2361 | **0.0481** | **0.0446** |
| 12.5, 50 | 0.7579 | 0.4744 | 0.4547 | 4.0860 | **0.0025** | **0.0009** | 4.4272 | **0.0023** | **0.0012** | 0.0000 | 1.0000 | 1.0000 | 3.2660 | **0.0100** | **0.0076** |
| 12.5, 100 | 0.1974 | 0.8965 | 0.8459 | 7.5498 | **0.0003** | **0.0001** | 5.4174 | **0.0007** | **0.0003** | 2.7775 | **0.0337** | **0.0161** | 6.3509 | **0.0005** | **0.0001** |
| 25, 37.5 | 0.9869 | 0.3665 | 0.3427 | 1.9640 | 0.0511 | 0.0775 | 0.6547 | 0.5596 | 0.5205 | 0.0000 | 1.0000 | 1.0000 | 0.3974 | 0.7177 | 0.7043 |
| 25, 50 | 1.3641 | 0.2139 | 0.1978 | 1.1275 | 0.2934 | 0.2847 | 2.5281 | **0.0273** | **0.0283** | 0.0000 | 1.0000 | 1.0000 | 1.4771 | 0.1757 | 0.1649 |
| 25, 100 | 0.8295 | 0.4398 | 0.4268 | 2.4495 | **0.0257** | **0.0309** | 3.4641 | **0.0084** | **0.0044** | 2.7775 | **0.0338** | **0.0168** | 3.3627 | **0.0073** | **0.0056** |
| 37.5, 50 | 0.3922 | 0.8359 | 0.6946 | 0.2774 | 0.7969 | 0.7823 | 1.6667 | 0.1251 | 0.1255 | 0.0000 | 1.0000 | 1.0000 | 1.0000 | 0.3355 | 0.3301 |
| 37.5, 100 | 0.0000 | 1.0000 | 1.0000 | 1.0000 | 0.3907 | 0.3378 | 2.3238 | **0.0383** | **0.0383** | 2.7775 | **0.0336** | **0.0155** | 2.4495 | **0.0350** | **0.0316** |
| 50, 100 | 0.3303 | 0.8597 | 0.7411 | 0.7924 | 0.5004 | 0.4459 | 0.4472 | 0.7078 | 0.6561 | 2.7775 | **0.0334** | **0.0197** | 1.1339 | 0.2858 | 0.2811 |
